# Hyperglycemia Aggravates Diet-Induced Coronary Artery Disease and Myocardial Infarction in SR-B1-Knockout/ApoE-Hypomorphic Mice

**DOI:** 10.3389/fphys.2018.01398

**Published:** 2018-10-09

**Authors:** Leticia Gonzalez, Melissa E. MacDonald, Yak D. Deng, Bernardo L. Trigatti

**Affiliations:** ^1^Thrombosis and Atherosclerosis Research Institute, McMaster University, Hamilton, ON, Canada; ^2^Department of Biochemistry and Biomedical Sciences, McMaster University, Hamilton, ON, Canada

**Keywords:** atherosclerosis, coronary artery, diabetes, fibrosis, hyperglycemia, myocardial infarction

## Abstract

Diabetes is a risk factor for development of atherosclerotic cardiovascular disease. Animal model studies in mice revealed that hyperglycemia increases development of atherosclerosis in the aorta as well as myocardial fibrosis in surgical models of coronary artery ligation; however, the impact of hyperglycemia on coronary artery atherosclerosis and subsequent heart disease is less clear. To investigate the effect of hyperglycemia on atherosclerosis and coronary heart disease, we used a mouse model of diet-induced coronary artery atherosclerosis and myocardial infarction, the high fat/high cholesterol (HFC) diet fed *SR-B1 knockout* (KO)*/apoE-hypomorphic* (HypoE) mouse. Hyperglycemia was induced in these mice by streptozotocin (STZ) treatment. This increased HFC diet-dependent atherosclerosis development (*p* = 0.02) and necrotic core formation (*p* = 0.0008) in atherosclerotic plaques in the aortic sinus but did not increase the extent of atherosclerosis in coronary arteries. However, it did increase the extent of platelet accumulation in atherosclerotic coronary arteries (*p* = 0.017). This was accompanied by increased myocardial fibrosis (*p* = 0.005) and reduced survival (*p* = 0.01) compared to control-treated, normoglycemic mice. These results demonstrate that STZ-treatment exerted differential effects on the level of atherosclerosis in the aortic sinus and coronary arteries. These results also suggest that SR-B1-KO/HypoE mice may be a useful non-surgical model of diabetic cardiomyopathy in the context of coronary artery atherothrombosis.

## Introduction

Type I diabetes patients present an increased risk of developing cardiovascular diseases relative to the general population (de Ferranti et al., 2014). Furthermore, coronary artery disease represents the leading cause of death among patients with diabetes mellitus (Secrest et al., 2010). In fact, children with type 1 diabetes exhibit increased aortic intima-media thickness, an early marker of subclinical atherosclerosis (Harrington et al., 2010). Studies in animal models, such as atherosclerosis susceptible mice, have revealed that hyperglycemia is associated with increased aortic sinus atherosclerosis (Park et al., 1998; Vikramadithyan et al., 2005; Werstuck et al., 2006; Johnson et al., 2011; Veerman et al., 2013; Venegas-Pino et al., 2013; Al-Sharea et al., 2018). However, the effects of hyperglycemia on coronary artery atherosclerosis are less well-studied, in part because conventional mouse atherosclerosis models, such as apolipoprotein E (apoE) or LDLR deficient mice, do not develop substantial coronary artery atherosclerosis or subsequent myocardial infarction (Gonzalez et al., 2016; Trigatti and Fuller, 2016).

Mice deficient in the HDL receptor, SR-B1, exhibit substantially increased HDL cholesterol as a result of impaired hepatic HDL cholesterol clearance (Rigotti et al., 1997). Mice deficient in both SR-B1 and apo E, *SR-B1/apoE* double KO mice, exhibit accelerated aortic sinus atherosclerosis development (Trigatti et al., 1999) as well as spontaneous development of extensive, occlusive atherosclerosis in coronary arteries, myocardial infarction, and early death (∼6–8 weeks of age; Braun et al., 2002). We and others reported similar results for mice deficient in both SR-B1 and the LDLR (*SR-B1/LDLR* double KO mice) fed HFC atherogenic diets (Fuller et al., 2014; Liao et al., 2017). *SR-B1* KO mice homozygous for a hypomorphic mutant apoE allele (*hypoE*) also develop HFC diet-induced coronary artery atherosclerosis, myocardial infarction, cardiac dysfunction, and reduced survival (Zhang et al., 2005; Nakagawa-Toyama et al., 2012; Hermann et al., 2016; Luk et al., 2016). Taking advantage of the inducible nature of the coronary artery atherosclerosis and myocardial infarction in *SR-B1-KO/hypoE* mice, we tested whether the induction of hyperglycemia in these mice affected the development of the diet induced coronary heart disease phenotype.

Hyperglycemia was induced by treatment of *SR-B1-KO/hypoE* mice with STZ. We report that multiple low dose STZ-treatment was not sufficient to induce coronary artery atherosclerosis in *SR-B1-KO/hypoE* mice fed a normal low fat/low cholesterol diet, and did not increase the extent of HFC diet induced coronary artery atherosclerosis. However, it did increase atherosclerotic plaque sizes and the sizes of necrotic cores in atherosclerotic plaques in the aortic sinus, and increased the extent of platelet accumulation in atherosclerotic coronary arteries in HFC diet fed *SR-B1-KO/hypoE* mice. Furthermore, STZ-treatment substantially increased cardiac fibrosis, and reduced the survival of HFC diet fed *SR-B1-KO/hypoE* mice. These findings suggest that STZ-induced hyperglycemia increased plaque thrombosis and myocardial infarction independently of alterations in atherosclerosis development in coronary arteries and suggest that HFC diet fed *SR-B1-KO/hypoE* mice may be a useful non-surgical model for hyperglycemia-induced plaque thrombosis and myocardial infarction.

## Materials and Methods

### Materials

Citrate buffer was purchased from Electron Microscopy Sciences (Hatfield, PA, United States). STZ and all other materials were purchased from Sigma Aldrich (St. Louis, MO, United States) unless indicated otherwise.

### Animals

All procedures were approved by the McMaster University Animal Research Ethics Board in accordance with the Canadian Council on Animal Care. Mice were housed in the Thrombosis and Atherosclerosis Research Institute (TaARI) animal facility in a *Helicobacter sp.* and murine noravirus positive room under controlled light (12 h light/dark) and temperature conditions. Mice were bred and housed in ventilated cages, had free access to food and received automatic watering. *SR-B1^-/-^*;*apoE-R61^hypomorphic^* (*SR-B1-KO/hypoE*) mice (mixed *C57BL/6:129* background) were produced by mating female *SR-B1^+/-^/hypoE* and male *SR-B1-KO/hypoE* mice from founders originally obtained from Monty Krieger (Massachusetts Institute of Technology, Cambridge, MA, United States; Zhang et al., 2005; Pei et al., 2013). Experimental mice were housed 2–4/cage. To induce diabetes, 5- or 8-weeks-old mice were given daily i.p. injections of STZ (40 mg/kg body weight in 20 μl) for 5 days, were rested for 1 week, and then received another series of five daily injections of STZ; control mice received vehicle (20 μl of 20 mmol/l citrate) following the same schedule (Werstuck et al., 2006). Non-fasting blood glucose was monitored weekly (see below). Mice with confirmed hyperglycemia were kept in the study, whereas mice whose blood glucose levels returned to baseline were removed. All mice in the study were males because we initially experienced difficulty in reproducibly inducing hyperglycemia in female mice. Mice were fed either normal rodent chow (Harlan Teklad TD2018, Madison, WI, United States) throughout the study, or, 3 weeks after initiation of STZ or control citrate buffer injection, mice were switched to a HFC diet (Harlan Teklad TD94059, Madison, WI, United States), containing 15.8% (by weight) fat and 1.25% (by weight) cholesterol. Mice that were to be fed the HFC diet were treated with STZ or control citrate buffer starting at 5 weeks of age, whereas mice that were maintained on normal chow throughout the study were treated with STZ or control citrate buffer beginning at 8 weeks of age. For survival studies, mice fed the HFC diet were monitored until they reached humane endpoint at which point they were euthanized (Fuller et al., 2014). Otherwise, mice fed the HFC diet were fasted for 4 h and sacrificed 7 weeks after the start of STZ- or control citrate buffer injection (4 weeks of HFC diet feeding) or, for mice maintained on normal chow diet, 14 weeks after the start of STZ- or control citrate buffer injection.

### Blood and Plasma Analysis

Plasma total cholesterol was measured using the Infinity Total Cholesterol assay kit (ThermoFisher Scientific, Burlington, ON, Canada). Unesterified cholesterol was measured using the Free Cholesterol E assay kit (Wako Diagnostics, Richmond VA, United States). Cholesteryl ester levels were calculated as the difference between total cholesterol and unesterified cholesterol measurements. HDL-cholesterol was measured with the HDL-Cholesterol E assay kit (Wako Diagnostics, Richmond VA, United States). Triglyceride was measured with the Infinity Triglycerides Liquid Stable assay kit (ThermoFisher Scientific, Burlington, ON, Canada). IL-6 and TNF-α were measured by ELISA (Biolegend, San Diego, CA, United States). Blood glucose was measured using a commercial glucometer (Contour Glucose Meter, Bayer). We observed that the high lipid levels in samples from mice fed the HFC diet appeared to interfere with the glucose measurements. To control for this, a standard curve was constructed by spiking plasma from HFC diet fed mice with known concentrations of added glucose.

### Histology

Cryosections (10 μm thick) from the top half (base) of the heart and the aortic sinus were stained with oil red O and hematoxylin, hematoxylin and eosin or Masson’s Trichrome, as previously described(Al-Jarallah et al., 2013; Pei et al., 2013; Fuller et al., 2014; Yu et al., 2018b). Aortic sinus atherosclerosis and coronary artery atherosclerosis burden were measured in oil red O and hematoxylin-stained sections as previously described (Al-Jarallah et al., 2013; Pei et al., 2013; Fuller et al., 2014; Yu et al., 2018b). Necrotic core sizes were measured as the a-nuclear and a-cellular areas within hematoxylin and eosin-stained plaques and were normalized to plaque area, as previously described (Gonzalez et al., 2017; Yu et al., 2018a). Myocardial fibrosis was detected in transverse sections of hearts with Masson’s Trichrome, which stains collagen-rich tissue blue and healthy myocardium red. Images of Trichrome-stained transverse heart sections are composites taken at 10 × magnification with an Olympus BX41 microscope and assembled using Slidebook 5.0 software. Percentage of myocardial fibrosis was quantified as previously described (Al-Jarallah et al., 2013; Pei et al., 2013; Fuller et al., 2014; Yu et al., 2018b).

### Immunofluorescence

Tissue sections were stained with rat anti-mouse CD41 antibody (catalog number 553847, BD Pharmingen, Mississauga, ON, Canada) followed by alexa 488-conjugated goat anti-rat IgG (catalog number A-11006, Invitrogen, ThermoFisher Scientific, Burlington, ON, Canada). Periostin was detected by immunofluorescence using a rabbit anti-periostin polyclonal antibody (catalog number ab92460, Abcam Inc., Toronto, ON, Canada) followed by alexa 488-conjugated goat anti-rabbit IgG (catalog number A-11008, Invitrogen, ThermoFisher Scientific, Burlington, ON, Canada). Sections were counterstained with DAPI to visualize nuclei. All images were acquired with a Zeiss Axiovert 200M inverted microscope with a 20 or 40x objective. CD41-positive coronary arteries were counted across five tissue sections. Results were expressed as the average number of CD41-positive coronary arteries per tissue section.

### Statistical Analysis

Results are presented as mean ± standard error of the mean (SEM). Survival curves were analyzed by the Mantel-Cox log-rank test. For comparison of two groups, data were subjected to the Mann–Whitney rank sum test as indicated. To analyze significant differences between more than two groups, one-way ANOVA followed by Tukey multiple comparisons test or two-way ANOVA followed by Sidak’s multiple comparisons *post hoc* test were used. Statistical analysis was performed using PRISM software (GraphPad Software Inc., La Jolla, CA, United States). *P* < 0.05 was considered to be significant.

## Results

### Effects of STZ-Treatment of *SR-B1-KO/hypoE* Mice on Hyperglycemia and Plasma Lipids

**FIGURE 1:**
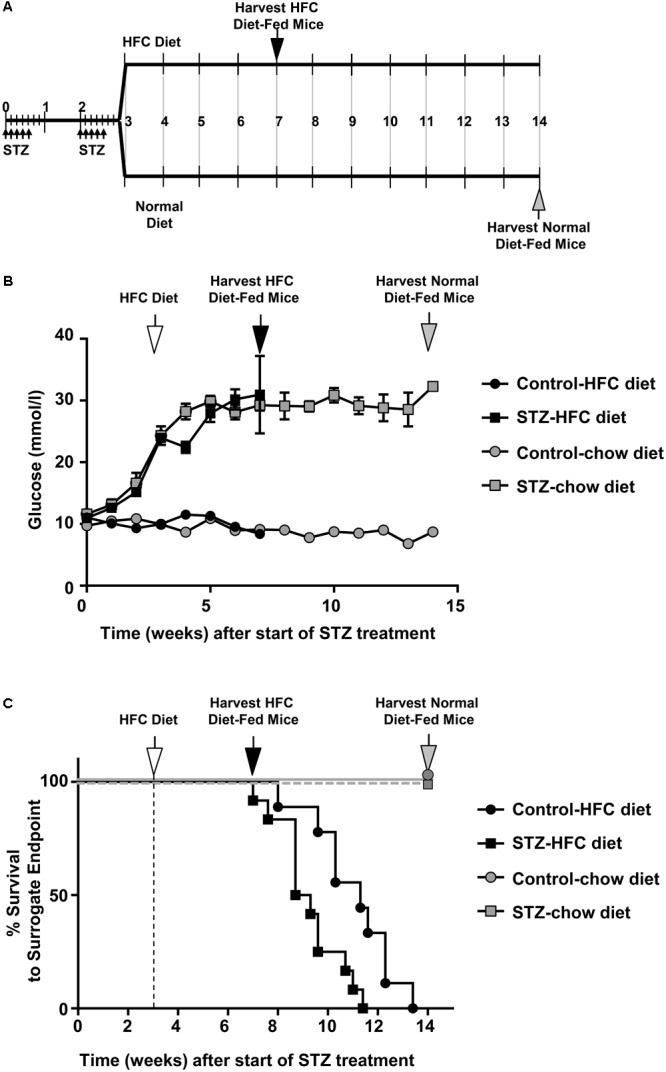
STZ-induced diabetes is associated with reduced survival HFC diet fed *SR-B1-KO/hypoE* mice. **(A)** Schematic representation of experimental time course. Male mice were treated with two rounds of low dose (40 mg/kg body weight) STZ injections (daily for 5 days each round) during weeks 1 and 3 (small arrows). Control mice received citrate buffer (not shown). At week 3, mice were fed either a HFC diet containing 15% fat and 1.25% cholesterol, or were maintained on a normal chow diet. Mice were either euthanized for analysis at week 7 (after 4 weeks of feeding the HFC diet; black arrow), week 14 (for normal chow diet; gray arrow) or were monitored for surrogate endpoint at which time they were humanly euthanized. **(B)** Non-fasting blood glucose levels over the course of the study for*(control- (circles) or STZ-treated mice (squares) fed either the normal chow (gray symbols) or switched to the HFC diet (white arrow; black symbols). Symbols represent means ± SEM of *n* = 7 (control-treated, fed normal chow), *n* = 6 (STZ-treated, fed normal chow), *n* = 14 (control-treated, fed HFC diet), and *n* = 15 (STZ-treated, fed HFC diet). Data were subjected to two-way ANOVA; *p* < 0.0001 for control- vs STZ-treated mice fed each diet, and for STZ-treated mice fed normal chow vs HFC diet. **(C)** Survival to surrogate endpoint for control- (circles) or STZ-treated mice (squares) fed either the normal chow diet (gray symbols) or the HFC diet (black symbols). The vertical dashed line and white arrow indicates the start of HFC diet feeding at 3 weeks (after start of STZ- or control citrate buffer treatment). *P* < 0.0001 for comparison between mice fed the normal chow and HFC diets and *p* = 0.009 for control- vs STZ-treated mice fed the HFC diet (by Mantel–Cox log-rank test).)*

The experimental scheme is shown in **Figure 1A**. *SR-B1-KO/hypoE* mice that were treated with STZ exhibited increased blood glucose levels beginning 2–3 weeks after the start of STZ injections (**Figure 1B**). Occasionally, we observed individual mice whose blood glucose levels returned to baseline levels after STZ treatment was discontinued; those were removed from the study. Blood glucose levels did not increase in *SR-B1-KO/hypoE* mice injected with citrate buffer vehicle (denoted as “control”; **Figure 1B**). We saw no differences in blood glucose levels between STZ-treated mice that had been maintained on a normal chow diet or mice that had been fed the HFC diet. Mice that were maintained on normal chow, regardless of treatment with STZ or control citrate buffer, exhibited no obvious clinical signs (other than dramatically increased urination in the STZ treated mice) and survived to the end of the study (14 weeks after initiation of STZ or control citrate buffer treatment; **Figure 1C**) at which point, they were euthanized for further analysis. *SR-B1-KO/hypoE* mice are known to develop HFC diet induced occlusive coronary artery atherosclerosis and fatal myocardial infarction (Zhang et al., 2005; Nakagawa-Toyama et al., 2012; Pei et al., 2013; Hermann et al., 2016; Luk et al., 2016). Consistent with this, *SR-B1-KO/hypoE* mice that had been treated with control citrate buffer and switched to the HFC diet exhibited reduced survival, with a median survival of 11.3 weeks after the start of treatment (8.3 weeks after the start of HFC diet feeding). The reduced survival of *SR-B1-KO/hypoE* mice that had been treated with STZ was even more pronounced, with a median survival of 9 weeks after the start of STZ treatment (6 weeks after the start of HFC diet feeding). Thus, STZ-treated *SR-B1-KO/hypoE* mice exhibited a greater reduction in survival in response to the HFC diet than control-treated mice.

**FIGURE 2:**
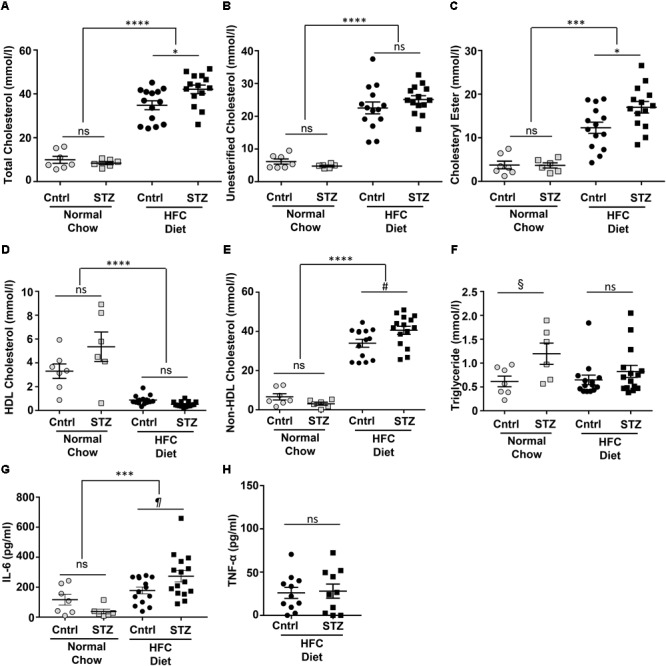
Plasma lipids and IL-6 in control- and STZ-treated *SR-B1-KO/hypoE* mice fed the normal chow or HFC diets. Mice were fasted for 4 h prior to humane euthanasia under anesthesia, and blood collection by cardiac puncture into heparinized syringes. Plasma was analyzed for total cholesterol **(A)**, unesterified cholesterol **(B)**, cholesteryl ester (total-unesterified cholesterol; **C**), HDL cholesterol **(D)**, non-HDL cholesterol (total – HDL cholesterol; **E)**, triglycerides **(F)** IL-6 **(G)**, and TNF-α **(H)** using commercially available assay kits as described in Section “Materials and Methods.” Circles represent mice treated with control citrate buffer (Cntrl) and squares represent mice treated with STZ. Gray symbols represent mice maintained on normal chow up to 14 weeks after start of control/STZ-treatment and black symbols represent mice switched to HFC diet 3 weeks after start of control/STZ-treatment and analyzed after 4 weeks of HFC diet feeding. Horizontal bars and error bars represent means and SEM. Data were analyzed by one-way ANOVA with the Tukey’s multiple comparisons test; ns indicates not statistically significant; ^∗^*p* < 0.05, ^∗∗∗^*p* < 0.001; ^∗∗∗∗^*p* < 0.00001. Pairwise comparisons indicated by the symbols #, §, and ¶were done using the Mann–Whitney rank sum test. ^#^*p* = 0.02; ^§^*p* = 0.051; ^¶^*p* = 0.061.

High fat/high cholesterol diet feeding significantly increased total and unesterified cholesterol, cholesteryl ester, and non-HDL cholesterol and reduced HDL cholesterol in plasma of *SR-B1-KO/hypoE* mice (**Figures 2A–E**). STZ-treatment did not affect plasma cholesterol levels in *SR-B1-KO/hypoE* mice maintained on normal chow (other than slightly increasing mean HDL cholesterol levels), but was associated with a 20% increase in average plasma levels of total cholesterol, and cholesteryl ester in *SR-B1-KO/hypoE* mice fed the HFC diet (**Figures 2A–E**). A similar trend toward increased mean non-HDL cholesterol was seen in STZ- vs control-treated mice fed the HFC diet but this did not reach statistical significance by one-way ANOVA (although it did appear to reach significance when only the control and STZ-samples for HFC diet fed mice were analyzed; **Figures 2A–E**). Similarly, trends toward increased triglyceride levels were observed in STZ-treated compared to control-treated mice. For mice maintained on the normal chow diet, these were just shy of significance (**Figure 2F**). HFC diet feeding was associated with statistically significantly increased plasma IL-6 levels in mice treated with STZ. For mice fed the HFC diet, there was a trend toward higher mean IL-6 levels for STZ- compared to control-treated mice (although this did not reach statistical significance; **Figure 2G**). No differences were detected in plasma TNF-α levels between STZ- and control-treated mice fed the HFC diet (**Figure 2H**).

### Effect of STZ-Treatment on HFC Diet-Induced Atherosclerosis in *SR-B1-KO/hypoE* Mice

**FIGURE 3:**
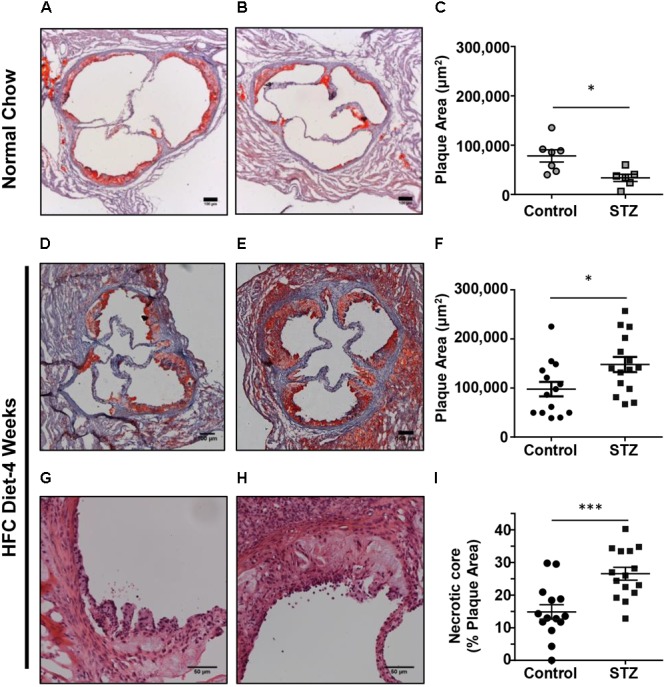
Effects of STZ-treatment on atherosclerosis in the aortic sinus. **(A,B)** Representative images of oil red O/hematoxylin-stained cross sections of the aortic sinus of control- and STZ-treated mice maintained on the normal chow diet up to 14 weeks after the start of STZ-treatment. Scale bars represent 100 μm. **(C)** Quantification of atherosclerotic plaque area (*n* = 7 control- and 6 STZ-treated mice; ^∗^*p* = 0.01). **(D,E)** Representative images of oil red O/hematoxylin-stained cross sections of the aortic sinus of control- and STZ-treated mice switched to the HFC diet 3 weeks after the start of STZ-treatment, and analyzed after 4 weeks of HFC diet feeding. Scale bars represent 100 μm. **(F)** Quantification of atherosclerotic plaque area (*n* = 14 control- and 15 STZ-treated mice; ^∗^*p* = 0.02). **(G,H)** Representative images of hematoxylin and eosin-stained atherosclerotic plaques in the aortic sinus of control and STZ-treated mice after 4 weeks of HFC diet feeding, showing necrotic cores devoid of nuclei and cells. Scale bars represent 50 μm. **(I)** Quantification of necrotic core area expressed as a percentage of the total plaque cross-sectional area per section (*n* = 14 control and 15 STZ treated mice; ^∗∗∗^*p* = 0.008). Data were analyzed by the Mann–Whitney rank sum test.

To examine the effects of STZ-treatment on atherosclerosis in *SR-B1-KO/hypoE* mice, we first examined atherosclerotic plaque sizes in the aortic sinus of mice maintained on a normal chow diet and euthanized 14 weeks after the start of STZ-treatment. Mice treated with control citrate buffer and then maintained on a normal chow diet developed atherosclerotic plaques in the aortic sinus, with an average ± SEM plaque cross-sectional area of 78,000 ± 12,000 μm^2^. Surprisingly, mice treated with STZ and maintained on a normal chow diet had smaller aortic sinus atherosclerotic plaque sizes with an average of 33,000 ± 7,000 μm^2^ (**Figures 3A–C**). When mice were fed the HFC diet beginning 3 weeks after the start of STZ or control citrate buffer treatment, and then euthanized for analysis at week 7 (4 weeks after the start of HFC diet feeding), the average atherosclerotic plaque cross-sectional area in the aortic sinus reached 97,600 ± 14,700 μm^2^ for mice treated with control citrate buffer and 145,000 ± 15,300 μm^2^ for mice treated with STZ (**Figures 3D–F**). Therefore, STZ-treatment was associated with a statistically significant, 50% increase in average diet-induced atherosclerosis in the aortic sinus of *SR-B1-KO/hypoE* mice. Similarly, STZ-treated mice fed the HFC diet exhibited a significant increase in the average necrotic core size (26.5 ± 1.9 vs 14.9 ± 2.2% of plaque size for STZ- vs control-treated mice that had been fed the HFC diet; **Figures 3G–I**). This is consistent with observations that STZ-treatment increases high fat diet-induced atherosclerosis in other mouse models such as low density receptor-deficient mice (Vikramadithyan et al., 2005; Johnson et al., 2011; Al-Sharea et al., 2018).

**FIGURE 4:**
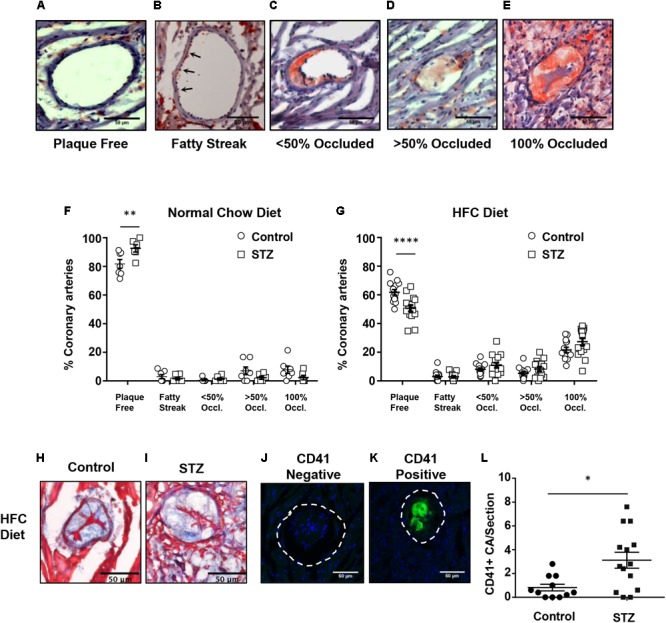
HFC diet-induced atherosclerosis and platelet accumulation in coronary arteries of control and STZ-treated *SR-B1-KO/hypoE* mice. Heart cross sections were stained with oil red O and hematoxylin. **(A–E)** Representative images of coronary arteries classified as having no atherosclerotic plaques (“plaque free”), fatty streaks (arrows), identified as oil red O staining within the wall without the presence of raised plaque, or containing raised atherosclerotic plaques occluding <50, >50, or 100% of the artery lumen (as shown). Quantification of the average proportions of coronary arteries per section classified according to the degree of occlusion in control- (circles) or STZ-treated mice (squares) **(F)** maintained on normal chow diet for 14 weeks after the start of control- or STZ-treatment (*n* = 7 and 6, respectively); or **(G)** switched to the HFC diet 3 weeks after start of treatment and analyzed after 4 weeks of HFC diet feeding (*n* = 14 and 15, respectively). Representative images of trichrome stained occluded coronary arteries from control- **(H)** or STZ-treated mice **(I)** fed the HFC diet for 4 weeks. Representative images of atherosclerotic CA’s stained for activated platelets (CD41, green) and nuclei (DAPI, blue) showing an atherosclerotic coronary artery that is negative **(J)** and an atherosclerotic coronary artery that is positive **(K)** for CD41 staining. The dashed line represents the vessel wall. **(L)** Quantification of the numbers of CD41^+^ atherosclerotic coronary arteries per section for control-treated (*n* = 11, circles) and STZ-treated mice that had been fed the HFC diet for 4 weeks (*n* = 14, squares). Scale bars represent 50 μm. Data in **F** and **G** were analyzed by two-way ANOVA with Sidak’s multiple comparisons test. Data in **L** were analyzed by the Mann–Whitney rank sum test. ^∗^*p* = 0.017; ^∗∗^*p* < 0.01, ^∗∗∗∗^*p* < 0.0001. All other comparisons between control- and STZ-treated samples from **F** and **G** were not statistically significantly different.

*SR-B1-KO/hypoE* mice fed the HFC diet develop atherosclerosis in coronary arteries in addition to the aortic sinus (Zhang et al., 2005; Nakagawa-Toyama et al., 2012; Pei et al., 2013; Hermann et al., 2016; Luk et al., 2016). We therefore examined the effects of STZ- vs control-treatment on atherosclerosis in coronary arteries in *SR-B1-KO/hypoE* that had been maintained on chow diet until 14 weeks after the induction of STZ or control- treatment, and in STZ- or control-treated *SR-B1-KO/hypoE* mice that had been fed the HFC diet beginning 3 weeks after the start of STZ- or control-treatment for a total of 4 weeks of HFC diet feeding (**Figures 4A–G**). To evaluate the extent of coronary artery atherosclerosis, coronary arteries were identified in oil red O/hematoxylin stained sections from the base of the aortic sinus to the midpoint of the heart and the numbers of coronary arteries exhibiting either no atherosclerosis, evidence of fatty streaks, or atherosclerotic plaques occluding less than 50%, greater than 50%, or 100% of the lumen of the coronary artery (**Figures 4A–E**) were counted. STZ- or control-treated mice maintained on normal chow until 14 weeks after the start of STZ-treatment exhibited few atherosclerotic coronary arteries, with >80–90% of coronary arteries exhibiting no atherosclerosis (**Figure 4F**). In contrast, STZ- and control-treated mice that had been fed the HFC diet for 4 weeks before analysis exhibited numerous atherosclerotic coronary arteries, with on average 60 and 50% of coronary arteries per section exhibiting no atherosclerosis and on average 22 and 27% of coronary arteries per section exhibiting full occlusion of their lumen (**Figure 4G**). Surprisingly, STZ-treatment appeared to slightly reduce the small number of atherosclerotic and slightly increase the number of non-atherosclerotic coronary arteries in chow fed mice, while it slightly increased the number of atherosclerotic and slightly reduced the number of non-atherosclerotic coronary arteries in the mice fed the HFC diet. Nevertheless, the effect of STZ-treatment on the number of fully occluded coronary arteries in the HFC diet fed mice appeared to be modest and did not reach statistical significance (although the reduction in the number of coronary arteries that were not atherosclerotic did). Therefore, STZ-treatment was associated with only a modest effect on coronary artery atherosclerosis in HFC diet fed mice. We detected no apparent differences in the appearance of fully occluded, atherosclerotic coronary arteries either upon oil red O/hematoxylin staining (not shown) or trichrome staining (**Figures 4H,I**). However, immunostaining for CD41, a marker of activated platelets (**Figures 4J,K**), revealed that there was a greater number of occluded coronary arteries that were CD41-positive from STZ-treated compared to control citrate buffer-treated *SR-B1-KO/hypoE* mice that had been fed the HFC diet for 4 weeks (**Figure 4L**). This suggested increased platelet accumulation in atherosclerotic coronary arteries from the STZ-treated mice fed the HFC diet.

### STZ-Treatment Increases HFC Diet-Induced Cardiac Fibrosis in *SR-B1-KO/hypoE* Mice

**FIGURE 5:**
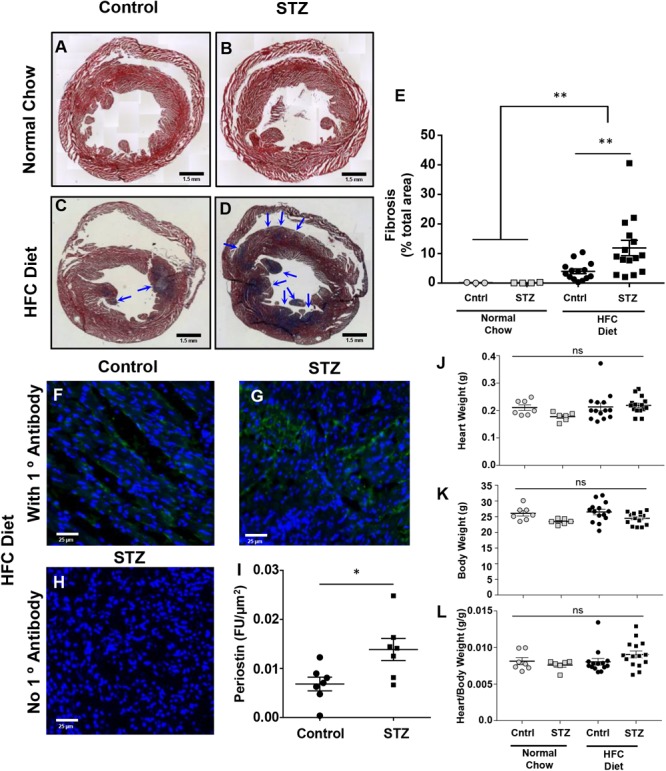
Effects of STZ- treatment on myocardial fibrosis in HFC diet fed *SR-B1-KO/hypoE* mice. Representative composite images of cardiac cross sections from **(A)** control- and **(B)** STZ-treated mice fed the normal chow diet and analyzed 14 weeks after the start of treatment, or **(C)** control- and **(D)** STZ-treated mice fed the HFC diet beginning 3 weeks after the start of treatment and analyzed after 4 weeks of HFC diet feeding. Sections are stained with Mason’s trichrome – healthy myocardium stains red and collagen stains blue (arrows). Scale bars represent 1.5 mm. **(E)** Quantification of average fibrotic (blue) area per section. Representative images of periostin staining (green) of cardiac sections from **(F)** control- and **(G)** STZ-treated mice fed the HFC diet beginning 3 weeks after the start of treatment and analyzed after 4 weeks of HFC diet feeding. **(H)** Control section of heart from an STZ-treated mouse fed the HFC diet in which the primary anti-periostin antibody was left out. Sections were counterstained with DAPI (blue). Scale bars represent 25 μm. **(I)** Quantification of periostin staining intensity per cardiac cross-sectional area for control- or STZ-treated mice that had been fed the HFC diet. **(J)** Heart weights, **(K)** body weights, and **(L)** heart/body weight ratios for control-treated (circles) and STZ-treated mice (squares) either maintained on the normal chow diet for 14 weeks after control/STZ-treatment (gray symbols) or fed the HFC diet for 4 weeks, beginning 3 weeks after control/STZ-treatment (black symbols). Each symbol in **E** and **I–L** represents an individual mouse. Means ± SEM are indicated by the horizontal lines and error bars. Data in **E** and **J–L** were analyzed by one-way ANOVA with Tukey’s multiple comparisons test and data in **I** were analyzed by the Mann–Whitney rank sum test; ns indicates not statistically significantly different (*p* > 0.05; ^∗^*p* = 0.018; ^∗∗^*p* = 0.005).

Trichrome staining of cardiac sections revealed that neither STZ- nor control-treated mice maintained on the normal chow diet exhibited detectable cardiac fibrosis (**Figures 5A,B,E**). In contrast, both control- and STZ-treated mice that had been fed the HFC diet for 4 weeks before sacrifice exhibited substantial cardiac fibrosis evident as the purple-blue staining of collagen (healthy myocardial tissue stains red; **Figures 5C–E**). A greater extent of cardiac fibrosis (measured as the percentage of the cardiac cross section that was stained blue) was seen in the HFC diet fed mice that had been treated with STZ compared to the control, citrate buffer (11.9 ± 2.6 vs 4.0 ± 0.8% of the cross-sectional area stained for collagen, **Figure 5E**). Immunostaining for periostin, a matricellular protein that regulates cardiac fibrosis (Frangogiannis, 2012; Landry et al., 2018) – revealed increased levels in the myocardial tissue of STZ- compared to control-treated *SR-B1-KO/hypoE* mice that were fed the HFC diet (**Figures 5F–I**). Despite the increased fibrosis, neither heart weights nor heart/body weight ratios were significantly different between the STZ- or control-treated mice fed either the normal chow or the HFC diet (**Figures 5J–L**). This suggests that the 4 week HFC feeding period may not have been long enough for the development of cardiomegaly, consistent with previous observations that cardiomegaly appears to develop after cardiac fibrosis and later in the disease process, closer to the sudden death of these mice (Pei et al., 2013).

## Discussion

*SR-B1-KO* mice containing atherogenic mutations in either apoE (*KO* or hypomorphic) or LDLR (*LDLR-KO*) develop either spontaneous (in the case of SR-B1/apoE double KO) or HFC diet-induced (in the case of *SR-B1-KO/hypoE* or *SR-B1/LDLR double KO* mice) coronary artery atherosclerosis, myocardial infarction, and dramatically reduced survival (Braun et al., 2002, 2003; Zhang et al., 2005; Karackattu et al., 2006; Nakagawa-Toyama et al., 2012; Al-Jarallah et al., 2013; Fuller et al., 2014; Hermann et al., 2016; Luk et al., 2016; Liao et al., 2017). The reduced survival of these mice appears to be associated with myocardial infarction and resulting cardiac conduction and functional abnormalities (Braun et al., 2002, 2003; Zhang et al., 2005; Karackattu et al., 2006; Nakagawa-Toyama et al., 2012; Al-Jarallah et al., 2013; Fuller et al., 2014; Hermann et al., 2016; Luk et al., 2016; Liao et al., 2017). In the case of the *SR-B1-KO/hypoE* and *SR-B1/LDLR double KO* mice, the timing of the onset of these phenotypes is dependent on the composition of the diet, with respect to fat and cholesterol and other components (such as cholate) which affect the diet’s atherogenicity (Nakagawa-Toyama et al., 2012; Fuller et al., 2014). Hyperglycemia is a risk factor for atherosclerotic disease in humans (Harrington et al., 2010; Secrest et al., 2010; de Ferranti et al., 2014), and has been shown to accelerate either spontaneous (*apoE-KO* mice), or diet-induced atherosclerosis (both *apoE-KO* and *LDLR-KO* mice) (Park et al., 1998; Vikramadithyan et al., 2005; Werstuck et al., 2006; Johnson et al., 2011; Veerman et al., 2013; Venegas-Pino et al., 2013; Al-Sharea et al., 2018). We therefore set out to test if hyperglycemia, alone, was sufficient to trigger coronary artery atherosclerosis development and myocardial infarction in *SR-B1-KO/hypoE* mice maintained on a normal chow diet, or to increase the onset of these phenotypes in these mice fed the HFC diet. To induce hyperglycemia, we exposed the mice to multiple low doses of STZ. STZ-treatment alone, in the absence of HFC diet, was not sufficient to trigger the development of coronary artery atherosclerosis (**Figure 4F**) or associated phenotypes, such as myocardial infarction (**Figures 5A,B,E**) and reduced survival (**Figure 1B**), at least within the 14 week timeframe of our study. By comparison, reduced survival of the control-treated *SR-B1-KO/hypoE* mice fed the HFC diet was seen as early as 5 weeks after the start of HFC diet feeding, with the median survival being 8.3 weeks after the start of HFC diet feeding (**Figure 1B**).

Surprisingly, we saw that STZ-treatment was associated with a reduction in spontaneous atherosclerosis development as measured by cross-sectional area of atherosclerotic plaques in the aortic sinus of *SR-B1-KO/hypoE* mice fed the normal chow diet. This is surprising given the literature that demonstrates that STZ-treatment generally increases spontaneous and high fat diet-induced atherosclerosis in *apoE-KO* and/or *LDLR-KO* mice (Park et al., 1998; Vikramadithyan et al., 2005; Werstuck et al., 2006; Johnson et al., 2011; Veerman et al., 2013; Venegas-Pino et al., 2013; Al-Sharea et al., 2018). It has, however, been reported that STZ-mediated induction of aortic sinus atherosclerotic plaque development in *apoE-KO* mice appears to be time dependent, with STZ-treatment exhibiting more pronounced effects early in atherosclerosis development and diminished effects with increased time (Veerman et al., 2013). Therefore, it is possible that the apparent protection against aortic sinus atherosclerosis observed by STZ-treatment in the normal chow diet fed *SR-B1-KO/hypoE* mice may be a feature of the timing of our analysis with respect to the start of STZ-treatment. Alternatively, we cannot rule out the possibility that this may represent unanticipated effects of STZ-treatment (e.g., other than the induction of hyperglycemia) or a unique response of these *SR-B1-KO/hypoE* mice to STZ-treatment.

Notwithstanding the above observations, we did observe that STZ-treatment was associated with increased sizes of aortic sinus atherosclerotic plaques and of necrotic cores within those plaques in the *SR-B1-KO/hypoE* mice fed the HFC diet (**Figure 3**). This is in agreement with previous reports of the effects of STZ and other approaches to induction of hyperglycemia in other mouse models of diet-induced atherosclerosis (Vikramadithyan et al., 2005; Johansson et al., 2008; Johnson et al., 2011; Al-Sharea et al., 2018; Venegas-Pino et al., 2018). Surprisingly, STZ-induced hyperglycemia did not substantially affect the burden of coronary artery atherosclerosis development in the HFC diet fed *SR-B1-KO/hypoE* mice, measured as the numbers of atherosclerotic coronary arteries (**Figure 4G**). However, it was associated with substantially increased platelet accumulation in the atherosclerotic coronary arteries of the HFC diet fed *SR-B1-KO/hypoE* mice (**Figures 4J–L**). This is in agreement with studies demonstrating increased incidence of coronary thrombosis in diabetic patients (Silva et al., 1995). STZ-treated mice fed the HFC diet also exhibited more extensive myocardial fibrosis as detected by trichrome staining (**Figures 5C–E**). Consistent with increased fibrosis, they exhibited increased abundance of periostin (**Figures 5F–I**), a transforming growth factor β-inducible matricellular protein known to play an important role in cardiac fibrosis and induced post-myocardial infarction and in diabetic cardiomyopathy (Frangogiannis, 2012; Guan et al., 2015; Landry et al., 2018). Periostin has been reported to be induced in response to myocardial infarction and during diabetic cardiomyopathy and to play an important signaling role in driving cardiac fibrosis (Frangogiannis, 2012; Guan et al., 2015; Landry et al., 2018). The observation that STZ-treated mice exhibit increased periostin staining in their myocardium compared to control-treated mice in response to HFC diet feeding is consistent with enhanced cardiac remodeling in response to hyperglycemia. Diabetes (both types 1 and 2) has been shown to induce diabetic cardiomyopathy, including enhancing myocardial fibrosis in rodent models of coronary artery ligation-induced ischemia/reperfusion injury (Greer et al., 2006; Eguchi et al., 2012a,b; Li et al., 2012). Thus, it is possible that the increased cardiac fibrosis seen in STZ- compared to control-treated *SR-B1-KO/hypoE* mice fed the HFC diet may reflect a direct effect of hyperglycemia on cardiac fibrosis.

Alternatively, the increased coronary artery thrombosis may have contributed to the increased cardiac fibrosis. For example, Hermann et al. (2016) recently reported that coronary arteries from *SR-B1-KO/hypoE* mice, fed a more atherogenic diet containing cholate, exhibited features of plaque rupture, including intraluminal thrombi and pro-inflammatory phenotypes, and that their distribution corresponded to regions of hearts exhibiting infarction. Administration of acetylsalicylic acid to those mice resulted in no changes in the burden of coronary artery atherosclerosis but a significantly reduced coronary artery inflammation and thrombosis and myocardial infarction and extended survival of the mice (Hermann et al., 2016). In an analogous manner, the increased coronary artery thrombosis detected in the STZ- compared to control-treated mice fed the HFC diet may have contributed to the increased cardiac fibrosis and their reduced survival.

We have previously shown that platelet accumulation in atherosclerotic coronary arteries and myocardial fibrosis were both reduced in *SR-B1/apoE* dKO mice treated with the hydroxymethylglutaryl coenzyme A reductase inhibitor, rosuvastatin (Yu et al., 2018b). In that study, however, we found that rosuvastatin-treatment had additional effects other than reducing platelet accumulation in atherosclerotic coronary arteries; these included reducing atherosclerotic plaque development in the aortic sinus and coronary arteries and reducing the accumulation of oxidized lipids in both the aortic sinus and coronary arteries. Rosuvastatin did not, however, reduce the hypercholesterolemia in the *SR-B1/apoE* dKO mice; instead, plasma total cholesterol levels were, unexpectedly, substantially increased by rosuvastatin treatment; however, the reasons for this effect remain unclear (Yu et al., 2018b). In the present study, we found that STZ-treatment increased the mean plasma total cholesterol levels in HFC diet fed *SR-B1-KO/hypoE* mice by approximately 20% (**Figure 2A**). This appeared to be due to increased levels of cholesterol ester (**Figure 2C**) and largely reflected similar trends towards increased cholesterol in the non-HDL fraction. STZ-treatment was also associated with trends towards increased triglyceride levels in normal chow and HFC-diet fed mice, and IL-6, but not TNF-α in the HFC diet fed mice; however, these did not reach statistical significance (**Figures 2F,G**). The STZ-induced increase in plasma cholesterol levels in the HFC diet fed mice may also have contributed to the increased aortic sinus atherosclerosis, coronary artery plaque thrombosis, and/or myocardial fibrosis.

Streptozotocin-treated *SR-B1-KO/hypoE* mice also exhibited reduced survival upon HFC diet feeding, as compared to control-treated mice fed the same diet (**Figure 1C**). The early death of *SR-B1-KO/hypoE* mice fed atherogenic HFC diets and of related *SR-B1/apoE double KO* mice fed normal chow diets has been attributed to their striking cardiovascular phenotypes of coronary artery atherosclerosis and myocardial infarction, which are accompanied by cardiac conductance abnormalities and reduced left ventricular function (Braun et al., 2002, 2003; Karackattu et al., 2006; Al-Jarallah et al., 2013; Hermann et al., 2016; Luk et al., 2016; Liao et al., 2017). Therefore, we think that the earlier onset of reduced survival of STZ- compared to control-treated mice when fed the HFC diet (median survival of 6 vs 8.3 weeks, after the start of HFC feeding; **Figure 1C**) most likely reflects the greater severity/earlier onset of coronary artery atherothrombosis (**Figures 4J–L**) and myocardial fibrosis (**Figures 5A–I**). However, we cannot rule out the possibility that STZ-treatment may have had other, unanticipated and currently unknown effects, either independent of hyperglycemia or on other organ systems, that affected the survival of HFC diet fed *SR-B1-KO/hypoE* mice. On the other hand, it was recently reported that genetic induction of hyperglycemia (due to mutation of the insulin2 gene) in male *apoE-KO* mice resulted in coronary artery atherosclerosis, myocardial fibrosis, and reduced survival upon feeding a high fat, atherogenic diet (Venegas-Pino et al., 2018). The similarity in phenotypes (increased coronary artery disease, myocardial infarction, and reduced survival) across two different mouse models and modes of hyperglycemia induction (high fat diet fed *Ins2-akita*/*apoE KO* mice in Venegas-Pino et al., 2018 and HFC diet fed, STZ-treated *SR-B1-KO/hypoE* mice in this study) suggest that these effects are likely driven by the hyperglycemia.

## Conclusion

Our observations that STZ-treatment increased HFC diet induced coronary artery atherothrombosis, myocardial infarction, and early death of SR-B1-KO/apoE-hypo mice suggests that this may be a useful model to study the effects of hyperglycemia on diabetic cardiomyopathy that does not require surgical ligation of coronary arteries.

## Author Contributions

LG contributed to the experimental design, data collection, data analysis, and manuscript writing. MM contributed to animal care and glucose data collection. YD contributed to data collection. BT contributed to the experimental design, data analysis, and manuscript writing.

## Conflict of Interest Statement

BT has received past funding from AstraZeneca Inc. for unrelated research. BT is a co-inventor on a patent US6437215 B1 entitled “SR-BI and ApoE knockout animals and use thereof as models for atherosclerosis and heart attack.” The remaining authors declare that the research was conducted in the absence of any commercial or financial relationships that could be construed as a potential conflict of interest.

## Abbreviations

apo: apolipoprotein
DAPI: 4′,6′-diamidino-2-phenylindole
HDL: high density lipoprotein
HFC: high fat/high cholesterol
HypoE: apoE-hypomorphic
IL-6: interleukin-6
KO: knockout
LDLR: low density lipoprotein receptor
SR-B1: scavenger receptor class b type 1
STZ: streptozotocin
TNFα: tumor necrosis factor α

## References

[B1] Al-Jarallah A. Igdoura F. Zhang Y. Tenedero C. B. White E. J. MacDonald M. E. (2013). The effect of pomegranate extract on coronary artery atherosclerosis in SR-BI/APOE double knockout mice. *Atherosclerosis* 228 80–89. 10.1016/j.atherosclerosis.2013.02.025 23528829

[B2] Al-Sharea A. Murphy A. J. Huggins L. A. Hu Y. Goldberg I. J. Nagareddy P. R. (2018). SGLT2 inhibition reduces atherosclerosis by enhancing lipoprotein clearance in Ldlr(-/-) type 1 diabetic mice. *Atherosclerosis* 271 166–176. 10.1016/j.atherosclerosis.2018.02.028 29518749 PMC7196281

[B3] Braun A. Trigatti B. L. Post M. J. Sato K. Simons M. Edelberg J. M. (2002). Loss of SR-BI expression leads to the early onset of occlusive atherosclerotic coronary artery disease, spontaneous myocardial infarctions, severe cardiac dysfunction, and premature death in apolipoprotein E-deficient mice. *Circ. Res.* 90 270–276. 11861414 10.1161/hh0302.104462

[B4] Braun A. Zhang S. Miettinen H. E. Ebrahim S. Holm T. M. Vasile E. (2003). Probucol prevents early coronary heart disease and death in the high-density lipoprotein receptor SR-BI/apolipoprotein E double knockout mouse. *Proc. Natl. Acad. Sci. U.S.A.* 100 7283–7288. 10.1073/pnas.1237725100 12771386 PMC165867

[B5] de Ferranti S. D. de Boer I. H. Fonseca V. Fox C. S. Golden S. H. Lavie C. J. (2014). Type 1 diabetes mellitus and cardiovascular disease: a scientific statement from the American Heart Association and American Diabetes Association. *Circulation* 130 1110–1130. 10.1161/CIR.0000000000000034 25114208

[B6] Eguchi M. Kim Y. H. Kang K. W. Shim C. Y. Jang Y. Dorval T. (2012a). Ischemia-reperfusion injury leads to distinct temporal cardiac remodeling in normal versus diabetic mice. *PLoS One* 7:e30450. 10.1371/journal.pone.0030450 22347376 PMC3275560

[B7] Eguchi M. Xu G. Li R. K. Sweeney G. (2012b). Diabetes influences cardiac extracellular matrix remodelling after myocardial infarction and subsequent development of cardiac dysfunction. *J. Cell. Mol. Med.* 16 2925–2934. 10.1111/j.1582-4934.2012.01613.x 22862852 PMC4393721

[B8] Frangogiannis N. G. (2012). Matricellular proteins in cardiac adaptation and disease. *Physiol. Rev.* 92 635–688. 10.1152/physrev.00008.2011 22535894 PMC4411042

[B9] Fuller M. Dadoo O. Serkis V. Abutouk D. MacDonald M. Dhingani N. (2014). The effects of diet on occlusive coronary artery atherosclerosis and myocardial infarction in scavenger receptor class B, type 1/low-density lipoprotein receptor double knockout mice. *Arterioscler. Thromb. Vasc. Biol.* 34 2394–2403. 10.1161/ATVBAHA.114.304200 25212235

[B10] Gonzalez L. Qian A. S. Tahir U. Yu P. Trigatti B. L. (2017). Sphingosine-1-phosphate receptor 1, expressed in myeloid cells, slows diet-induced atherosclerosis and protects against macrophage apoptosis in Ldlr KO mice. *Int. J. Mol. Sci.* 18:E2721. 10.3390/ijms18122721 29244772 PMC5751322

[B11] Gonzalez L. Yu P. Trigatti B. L. (2016). Mouse models of coronary artery atherosclerosis. *J. Cardiovasc. Disord.* 3:1021.

[B12] Greer J. J. Ware D. P. Lefer D. J. (2006). Myocardial infarction and heart failure in the db/db diabetic mouse. *Am. J. Physiol. Heart Circ. Physiol.* 290 H146–H153. 10.1152/ajpheart.00583.2005 16113078

[B13] Guan J. Liu W. Q. Xing M. Q. Shi Y. Tan X. Y. Jiang C. Q. (2015). Elevated expression of periostin in diabetic cardiomyopathy and the effect of valsartan. *BMC Cardiovasc. Disord.* 15:90. 10.1186/s12872-015-0084-3 26281830 PMC4539668

[B14] Harrington J. Pena A. S. Gent R. Hirte C. Couper J. (2010). Aortic intima media thickness is an early marker of atherosclerosis in children with type 1 diabetes mellitus. *J. Pediatr.* 156 237–241. 10.1016/j.jpeds.2009.08.036 19853860

[B15] Hermann S. Kuhlmann M. T. Starsichova A. Eligehausen S. Schafers K. Stypmann J. (2016). Imaging reveals the connection between spontaneous coronary plaque ruptures, atherothrombosis, and myocardial infarctions in HypoE/SRBI-/- mice. *J. Nucl. Med.* 57 1420–1427. 10.2967/jnumed.115.171132 27127225

[B16] Johansson F. Kramer F. Barnhart S. Kanter J. E. Vaisar T. Merrill R. D. (2008). Type 1 diabetes promotes disruption of advanced atherosclerotic lesions in LDL receptor-deficient mice. *Proc. Natl. Acad. Sci. U.S.A.* 105 2082–2087. 10.1073/pnas.0709958105 18252823 PMC2538884

[B17] Johnson L. A. Arbones-Mainar J. M. Fox R. G. Pendse A. A. Altenburg M. K. Kim H. S. (2011). Apolipoprotein E4 exaggerates diabetic dyslipidemia and atherosclerosis in mice lacking the LDL receptor. *Diabetes Metab. Res. Rev.* 60 2285–2294. 10.2337/db11-0466 21810592 PMC3161311

[B18] Karackattu S. L. Trigatti B. Krieger M. (2006). Hepatic lipase deficiency delays atherosclerosis, myocardial infarction, and cardiac dysfunction and extends lifespan in SR-BI/apolipoprotein E double knockout mice. *Arterioscler. Thromb. Vasc. Biol.* 26 548–554. 10.1161/01.ATV.0000202662.63876.02 16397139

[B19] Landry N. M. Cohen S. Dixon I. M. C. (2018). Periostin in cardiovascular disease and development: a tale of two distinct roles. *Basic Res. Cardiol.* 113:1. 10.1007/s00395-017-0659-5 29101484

[B20] Li C. J. Lv L. Li H. Yu D. M. (2012). Cardiac fibrosis and dysfunction in experimental diabetic cardiomyopathy are ameliorated by alpha-lipoic acid. *Cardiovasc. Diabetol.* 11:73. 10.1186/1475-2840-11-73 22713251 PMC3472273

[B21] Liao J. Guo X. Wang M. Dong C. Gao M. Wang H. (2017). Scavenger receptor class B type 1 deletion led to coronary atherosclerosis and ischemic heart disease in low-density lipoprotein receptor knockout mice on modified western-type diet. *J. Atheroscler. Thromb.* 24 133–146. 10.5551/jat.33019 27373983 PMC5305674

[B22] Luk F. S. Kim R. Y. Li K. Ching D. Wong D. K. Joshi S. K. (2016). Immunosuppression With FTY720 reverses cardiac dysfunction in hypomorphic ApoE mice deficient in SR-BI expression that survive myocardial infarction caused by coronary atherosclerosis. *J. Cardiovasc. Pharmacol.* 67 47–56. 10.1097/FJC.0000000000000312 26322923 PMC4703534

[B23] Nakagawa-Toyama Y. Zhang S. Krieger M. (2012). Dietary manipulation and social isolation alter disease progression in a murine model of coronary heart disease. *PLoS One* 7:e47965. 10.1371/journal.pone.0047965 23112879 PMC3480446

[B24] Park L. Raman K. G. Lee K. J. Lu Y. Ferran LJ Jr Chow W. S. (1998). Suppression of accelerated diabetic atherosclerosis by the soluble receptor for advanced glycation endproducts. *Nat. Med.* 4 1025–1031. 10.1038/2012 9734395

[B25] Pei Y. Chen X. Aboutouk D. Fuller M. T. Dadoo O. Yu P. (2013). SR-BI in bone marrow derived cells protects mice from diet induced coronary artery atherosclerosis and myocardial infarct4ion. *PLoS One* 8:e72492. 10.1371/journal.pone.0072492 23967310 PMC3742605

[B26] Rigotti A. Trigatti B. L. Penman M. Rayburn H. Herz J. Krieger M. (1997). A targeted mutation in the murine gene encoding the high density lipoprotein (HDL) receptor scavenger receptor class B type I reveals its key role in HDL metabolism. *Proc. Natl. Acad. Sci. U.S.A.* 94 12610–12615. 9356497 10.1073/pnas.94.23.12610 PMC25055

[B27] Secrest A. M. Becker D. J. Kelsey S. F. Laporte R. E. Orchard T. J. (2010). Cause-specific mortality trends in a large population-based cohort with long-standing childhood-onset type 1 diabetes. *Diabetes Metab. Res. Rev.* 59 3216–3222. 10.2337/db10-0862 20739685 PMC2992785

[B28] Silva J. A. Escobar A. Collins T. J. Ramee S. R. White C. J. (1995). Unstable angina. A comparison of angioscopic findings between diabetic and nondiabetic patients. *Circulation* 92 1731–1736. 7671354 10.1161/01.cir.92.7.1731

[B29] Trigatti B. Rayburn H. Vinals M. Braun A. Miettinen H. Penman M. (1999). Influence of the high density lipoprotein receptor SR-BI on reproductive and cardiovascular pathophysiology. *Proc. Natl. Acad. Sci. U.S.A.* 96 9322–9327. 10430941 10.1073/pnas.96.16.9322 PMC17781

[B30] Trigatti B. L. Fuller M. (2016). HDL signaling and protection against coronary artery atherosclerosis in mice. *J. Biomed. Res.* 30 94–100. 10.7555/JBR.30.20150079 26642235 PMC4820886

[B31] Veerman K. J. Venegas-Pino D. E. Shi Y. Khan M. I. Gerstein H. C. Werstuck G. H. (2013). Hyperglycaemia is associated with impaired vasa vasorum neovascularization and accelerated atherosclerosis in apolipoprotein-E deficient mice. *Atherosclerosis* 227 250–258. 10.1016/j.atherosclerosis.2013.01.018 23411040

[B32] Venegas-Pino D. E. Lagrotteria A. Wang P. W. Morphet J. Clapdorp C. Shi Y. (2018). Evidence of extensive atherosclerosis, coronary artery disease and myocardial infarction in the ApoE(-/-):Ins2( + /Akita) mouse fed a western diet. *Atherosclerosis* 275 88–96. 10.1016/j.atherosclerosis.2018.05.044 29879686

[B33] Venegas-Pino D. E. Wang P. W. Stoute H. K. Singh-Pickersgill N. A. Hong B. Y. Khan M. I. (2013). Sex-specific differences in an ApoE(-/-):Ins2( + /Akita) mouse model of accelerated atherosclerosis. *Am. J. Pathol.* 186 67–77. 10.1016/j.ajpath.2015.09.009 26597883

[B34] Vikramadithyan R. K. Hu Y. Noh H. L. Liang C. P. Hallam K. Tall A. R. (2005). Human aldose reductase expression accelerates diabetic atherosclerosis in transgenic mice. *J. Clin. Invest.* 115 2434–2443. 10.1172/JCI24819 16127462 PMC1190371

[B35] Werstuck G. H. Khan M. I. Femia G. Kim A. J. Tedesco V. Trigatti B. (2006). Glucosamine-induced endoplasmic reticulum dysfunction is associated with accelerated atherosclerosis in a hyperglycemic mouse model. *Diabetes Metab. Res. Rev.* 55 93–101. 16380481

[B36] Yu P. Qian A. S. Chathely K. M. Trigatti B. L. (2018a). PDZK1 in leukocytes protects against cellular apoptosis and necrotic core development in atherosclerotic plaques in high fat diet fed ldl receptor deficient mice. *Atherosclerosis* 276 171–181. 10.1016/j.atherosclerosis.2018.05.009 29853191

[B37] Yu P. Xiong T. Tenedero C. B. Lebeau P. Ni R. MacDonald M. E. (2018b). Rosuvastatin reduces aortic sinus and coronary artery atherosclerosis in SR-B1 (Scavenger Receptor Class B Type 1)/ApoE (Apolipoprotein E) double knockout mice independently of plasma cholesterol lowering. *Arterioscler. Thromb. Vasc. Biol.* 38 26–39. 10.1161/ATVBAHA.117.305140 29162602 PMC5757666

[B38] Zhang S. Picard M. H. Vasile E. Zhu Y. Raffai R. L. Weisgraber K. H. (2005). Diet-induced occlusive coronary atherosclerosis, myocardial infarction, cardiac dysfunction, and premature death in scavenger receptor class B type I-deficient, hypomorphic apolipoprotein ER61 mice. *Circulation* 111 3457–3464. 10.1161/CIRCULATIONAHA.104.523563 15967843

